# Identification of *ROBO2* as a Potential Locus Associated with Inhaled Corticosteroid Response in Childhood Asthma

**DOI:** 10.3390/jpm11080733

**Published:** 2021-07-28

**Authors:** Natalia Hernandez-Pacheco, Mario Gorenjak, Jiang Li, Katja Repnik, Susanne J. Vijverberg, Vojko Berce, Andrea Jorgensen, Leila Karimi, Maximilian Schieck, Lesly-Anne Samedy-Bates, Roger Tavendale, Jesús Villar, Somnath Mukhopadhyay, Munir Pirmohamed, Katia M. C. Verhamme, Michael Kabesch, Daniel B. Hawcutt, Steve Turner, Colin N. Palmer, Kelan G. Tantisira, Esteban G. Burchard, Anke H. Maitland-van der Zee, Carlos Flores, Uroš Potočnik, Maria Pino-Yanes

**Affiliations:** 1 Research Unit, Hospital Universitario N.S. de Candelaria, Universidad de La Laguna, Carretera General del Rosario 145, 38010 Santa Cruz de Tenerife, Spain; cflores@ull.edu.es; 2 Genomics and Health Group, Department of Biochemistry, Microbiology, Cell Biology and Genetics, Universidad de La Laguna, Avenida Astrofísico Francisco Sánchez s/n, Faculty of Science, Apartado 456, 38200 San Cristóbal de La Laguna, Spain; mdelpino@ull.edu.es; 3 CIBER de Enfermedades Respiratorias, Instituto de Salud Carlos III, Avenida de Monforte de Lemos, 5, 28029 Madrid, Spain; jesus.villar54@gmail.com; 4 Center for Human Molecular Genetics and Pharmacogenomics, Faculty of Medicine, University of Maribor, Taborska Ulica 8, 2000 Maribor, Slovenia; mario.gorenjak@um.si (M.G.); katja.repnik82@gmail.com (K.R.); vojko.berce@guest.arnes.si (V.B.); 5 The Channing Division of Network Medicine, Department of Medicine, Brigham & Women’s Hospital and Harvard Medical School, 75 Francis St, Boston, MA 02115, USA; cougarlj@gmail.com (J.L.); rekgt@channing.harvard.edu (K.G.T.); 6 Laboratory for Biochemistry, Molecular Biology, and Genomics, Faculty of Chemistry and Chemical Engineering, University of Maribor, Smetanova ulica 17, 2000 Maribor, Slovenia; 7 Department of Respiratory Medicine, Amsterdam UMC, University of Amsterdam, Meibergdreef 9, 1105 AZ Amsterdam, The Netherlands; s.j.vijverberg@amc.uva.nl (S.J.V.); a.h.maitland@amc.uva.nl (A.H.M.-v.d.Z.); 8 Division of Pharmacoepidemiology and Clinical Pharmacology, Faculty of Science, Utrecht University, Princetonplein 5, 3584 CC Utrecht, The Netherlands; 9 Department of Pediatric Respiratory Medicine and Allergy, Emma’s Children Hospital, Amsterdam UMC, University of Amsterdam, Meibergdreef 9, 1105 AZ Amsterdam, The Netherlands; 10 Department of Pediatrics, University Medical Centre Maribor, Ljubljanska Ulica 5, 2000 Maribor, Slovenia; 11 Department of Biostatistics, University of Liverpool, Crown Street, Liverpool L69 3BX, UK; a.l.jorgensen@liverpool.ac.uk; 12 Department of Medical Informatics, Erasmus University Medical Center, Dr. Molewaterplein 40, 3015 GD Rotterdam, The Netherlands; l.karimi@erasmusmc.nl (L.K.); k.verhamme@erasmusmc.nl (K.M.C.V.); 13 Department of Pediatric Pneumology and Allergy, University Children’s Hospital Regensburg (KUNO), Franz-Josef-Strauß-Allee 11, 93053 Regensburg, Germany; schieck.maximilian@mh-hannover.de (M.S.); Michael.Kabesch@barmherzige-regensburg.de (M.K.); 14 Department of Human Genetics, Hannover Medical School, Carl-Neuberg-Straße 1, 30625 Hannover, Germany; 15 Department of Medicine, University of California, San Francisco, CA 94143, USA; lessam21@gmail.com (L.-A.S.-B.); Esteban.Burchard@ucsf.edu (E.G.B.); 16 Department of Bioengineering and Therapeutic Sciences, University of California, 533 Parnassus Ave, San Francisco, CA 94143, USA; 17 Population Pharmacogenetics Group, Biomedical Research Institute, Ninewells Hospital, and Medical School, University of Dundee, Dundee DD1 9SY, UK; r.tavendale@dundee.ac.uk (R.T.); s.mukhopadhyay@bsms.ac.uk (S.M.); palmerc@me.com (C.N.P.); 18 Multidisciplinary Organ Dysfunction Evaluation Research Network, Research Unit, Hospital Universitario Dr. Negrín, Calle Barranco de la Ballena s/n, 35019 Las Palmas de Gran Canaria, Spain; 19 Keenan Research Center for Biomedical Science, Li Ka Shing Knowledge Institute, St Michael’s Hospital, 30 Bond St, Toronto, ON M5B 1W8, Canada; 20 Academic Department of Paediatrics, Brighton and Sussex Medical School, Royal Alexandra Children’s Hospital, 94 N-S Rd, Falmer, Brighton BN2 5BE, UK; 21 Department of Molecular and Clinical Pharmacology, Institute of Translational Medicine, University of Liverpool, 200 London Rd, Liverpool L3 9TA, UK; munirp@liverpool.ac.uk; 22 Department of Women’s and Children’s Health, University of Liverpool, Liverpool L69 3BX, UK; D.Hawcutt@liverpool.ac.uk; 23 Alder Hey Children’s Hospital, E Prescot Rd, Liverpool L14 5AB, UK; 24 Child Health, University of Aberdeen, King’s College, Aberdeen AB24 3FX, UK; s.w.turner@abdn.ac.uk; 25 Division of Pulmonary and Critical Care Medicine, Department of Medicine, Brigham and Women’s Hospital, and Harvard Medical School, 75 Francis St, Boston, MA 02115, USA; 26 Genomics Division, Instituto Tecnológico y de Energías Renovables (ITER), Polígono Industrial de Granadilla, 38600 Granadilla, Spain; 27 Instituto de Tecnologías Biomédicas (ITB), Universidad de La Laguna, Faculty of Health Sciences, Apartado 456, 38200 San Cristóbal de La Laguna, Spain

**Keywords:** childhood asthma, exacerbations, forced expiratory volume in one second, genome-wide association study, inhaled corticosteroids, single nucleotide polymorphism

## Abstract

Inhaled corticosteroids (ICS) are the most common asthma controller medication. An important contribution of genetic factors in ICS response has been evidenced. Here, we aimed to identify novel genetic markers involved in ICS response in asthma. A genome-wide association study (GWAS) of the change in lung function after 6 weeks of ICS treatment was performed in 166 asthma patients from the SLOVENIA study. Patients with an improvement in lung function ≥8% were considered as ICS responders. Suggestively associated variants (*p*-value ≤ 5 × 10^−6^) were evaluated in an independent study (*n* = 175). Validation of the association with asthma exacerbations despite ICS use was attempted in European (*n* = 2681) and admixed (*n* = 1347) populations. Variants previously associated with ICS response were also assessed for replication. As a result, the SNP rs1166980 from the *ROBO2* gene was suggestively associated with the change in lung function (OR for G allele: 7.01, 95% CI: 3.29–14.93, *p* = 4.61 × 10^−7^), although this was not validated in CAMP. *ROBO2* showed gene-level evidence of replication with asthma exacerbations despite ICS use in Europeans (minimum *p*-value = 1.44 × 10^−5^), but not in admixed individuals. The association of *PDE10A-T* with ICS response described by a previous study was validated. This study suggests that *ROBO2* could be a potential novel locus for ICS response in Europeans.

## 1. Introduction

Asthma is the most common chronic disease in childhood and causes a high burden on the quality of life of the patients and their families. In addition, asthma has an impact in economic terms on the healthcare system, school, and/or work absenteeism [1,2]. This is a complex respiratory disorder characterized by inflammation and reversible obstruction of airways [3], and with diverse manifestations of symptoms, such as wheeze, breathlessness, chest tightness, and cough [1].

Inhaled corticosteroids (ICS) are the most effective and widely prescribed asthma preventive medication [1,2,4,5]. Patients with asthma benefit from ICS therapy through decreased airway inflammation, improved pulmonary capacity, and reduced asthma-related symptoms and exacerbations [6]. Although ICS has demonstrated efficacy in improving symptoms in most children with asthma, between 30% and 40% do not have a complete response to ICS treatment. Furthermore, 10–15% of the children treated with ICS may experience asthma exacerbations worsening or even suffer severe adverse effects [4,7]. Furthermore, not only interindividual differences in ICS response have been described, but also differences among different populations and ethnic groups [4,8]. These differences have been evidenced to be the result of the interaction of several factors, including comorbidities, environmental exposures, and the clinical characteristics of the disease, among others [9]. For instance, the type of asthma (e.g., atopic or non-atopic) could partly contribute to the responsiveness to ICS therapy in some patients. Therefore, subjects with atopic asthma and high levels of blood eosinophils could experience a greater benefit from ICS in contrast to those with a neutrophilic pattern [10,11]. Nonetheless, the important contribution of the individual’s genetic composition has also been suggested [12,13].

Different clinical markers that have been commonly used to evaluate ICS response include the asthma control test [14], asthma symptoms scores [15,16], information about exacerbations [17,18,19], and change in lung function after therapy [1,20]. Among these, performing serial measurements of lung function after a short period of therapy is the most commonly used marker for assessing treatment response [6,18]. The difference between forced expiratory volume in one second (FEV_1_) values measured at the beginning of treatment and a few weeks [21,22] or months [23] later provides substantial information about ICS response [23]. Importantly, the change in FEV_1_ after 6 weeks of treatment with ICS has been proposed to be a good predictor of long-term asthma control [21,22]. Although some limitations have been attributed to the evaluation of the lung function (e.g., measurement variability during the day, experience and potential errors driven by the operator, type and calibration of the equipment, and the interpretative algorithm), this approach provides a quantitative and objective measure of the response to asthma treatment [24,25]. Some authors have suggested that variability in ICS response may be explained by the interaction of several factors, including the individual’s genetic composition by means of heritability estimates [9,26]. It has been suggested that approximately 60–80% of the total variation in asthma treatment response might be explained by genetic factors [17,27,28].

Pharmacogenetic studies of ICS have been recently carried out mostly using the genome-wide association study (GWAS) approach [27]. To date, a total of twelve published GWASs have explored the association with ICS response mostly in European populations [28,29,30,31,32,33,34,35,36,37,38,39]. These have identified the association of 28 genetic variants located within or near 17 genes with different measurements of ICS response, being the most common definition of the change in FEV_1_ after a short period of treatment with ICS. Nonetheless, the validation of some of these associations has suggested that the assessment of the history of recent asthma exacerbations despite ICS treatment can also be used as a proxy of asthma treatment response in different populations [38,39]. Despite the effort of these studies, the genes identified do not explain the response to ICS treatment. Thus, these have not yet provided real improvements in the clinical strategies of asthma management [40], and further genetic variants are expected to be involved in ICS responsiveness [12].

Here, we conducted a pilot study to identify novel genetic variants associated with the response to ICS treatment by means of a GWAS of the change in FEV_1_ after initiating ICS therapy in asthma patients of European descent. Association with asthma exacerbations of the markers identified was attempted in children and youths treated with ICS from different populations.

## 2. Results

### 2.1. Characteristics of the Study Populations

One hundred sixty-six children and young adult asthma patients from the SLOVENIA study [38,39,41] with reported use of any ICS in the last 12 months were included in the discovery phase (Table 1). Of these, 94 were ICS non-responders (cases) and 72 were responders (controls), based on a threshold of 8% FEV_1_ improvement after 6 weeks of ICS therapy. The individuals included were 10.9 ± 3.4 years old on average, showing a similar mean age in both groups (cases: 10.7 ± 3.2 years, controls: 11.2 ± 3.5 years). ICS responders showed a substantial improvement in pulmonary capacity after 6 weeks of treatment with ICS (16.9% ± 8.7%).

Patients from the European and non-European studies included in the replication of results assessing the association with asthma exacerbations despite ICS treatment showed a similar mean age to those in the SLOVENIA study. However, followMAGICS included older participants (17.2 ± 3.0 years). Since asthma exacerbations were differentially defined among studies, there was variation in the exacerbation rates, ranging from 11.0% in PACMAN to 66.4% in GALA II. Further details about the clinical and demographic characteristics of the populations included in the replication with asthma exacerbations despite ICS use can be found in previous publications [38,39,42].

### 2.2. Association Results of the Change in FEV1 after ICS Treatment

**Figure 1:**
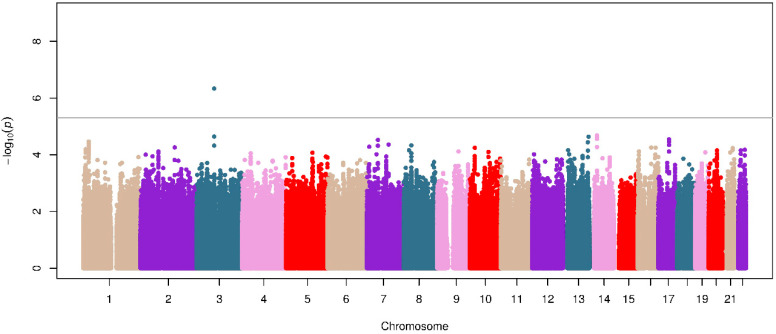
Manhattan plot of association results of the change in FEV_1_ after ICS treatment in SLOVENIA. The logarithmic transformation of the *p*-value (−log_10_ *p*-value) is represented on the *y*-axis, along with the chromosome position (*x*-axis). The gray line indicates the suggestive significance threshold (*p* ≤ 5 × 10^−6^) considered for evidence of association with ICS response.

**Figure 2:**
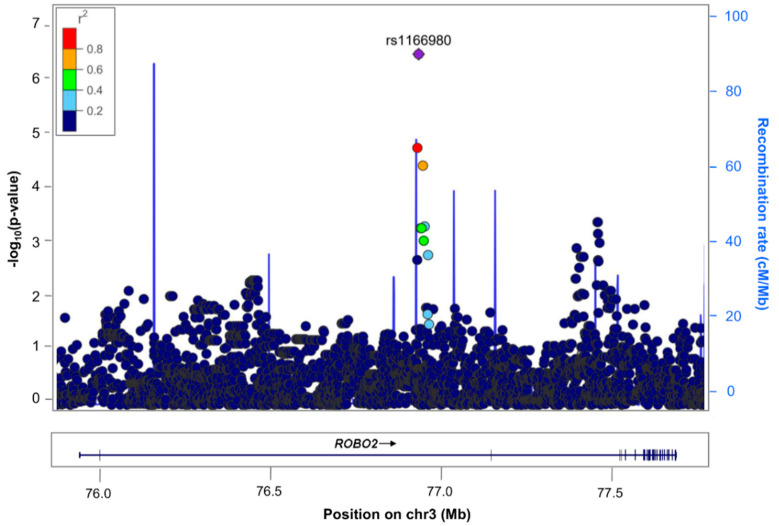
Regional plot of association results with the change in FEV_1_ after ICS treatment expressed as a binary variable in SLOVENIA. Results are represented based on the significance of the association on the *y*-axis (−log_10_ *p*-value) and chromosome position (*x*-axis) for each SNP as a dot. The SNP rs1166980 suggestively associated with ICS response is represented by the purple diamond. The remaining SNPs are color-coded based on pairwise linkage disequilibrium (*r*^2^ values) with that SNP for European populations from 1KGP (GRCh37/hg19 build).

A total of 7.5 million common single-nucleotide polymorphisms (SNPs) with minor allele frequency (MAF) ≥ 1% with an imputation quality (Rsq) ≥ 0.3 were tested for association with the binary outcome related to the change in FEV_1_ after ICS treatment in asthma patients from the SLOVENIA study. No evidence of genomic inflation due to population stratification effects was revealed by the value of λ_GC_ = 1.00 (Figure S1). No associations were found at genome-wide significance level (*p*-value ≤ 5 × 10^−8^), but the SNP rs1166980 located in the *ROBO2* gene was found to be suggestively (*p*-value ≤ 5 × 10^−6^) associated with ICS responsiveness in asthma patients (odds ratio (OR) for G allele: 7.01, 95% confidence interval (CI): 3.29–14.93, *p* = 4.61 × 10^−7^) (Figure 1 and Figure 2).

The association of rs1166980 was also found when assessing the quantitative measurement of the change in FEV_1_ in the same SLOVENIA participants, since the risk allele for non-response to ICS was also associated with lower lung function improvement (β for G allele: −6.54, 95% CI: −9.74–−3.34, *p* = 9.41 × 10^−5^). However, no evidence of association was found for the SNP rs1166980 when the binary (OR for G allele: 1.23, 95% CI: 0.69–1.77, *p* = 0.453) or the quantitative variables (β for G allele: −0.03, 95% CI: −0.07–0.01, *p* = 0.144) of the change in FEV_1_ after 2 months of ICS use were tested for association in the independent study Childhood Asthma Management Program (CAMP) (Table S1).

### 2.3. Validation of the Association with Asthma Exacerbations despite ICS Use

The association of the SNP rs1166980 was not replicated in Europeans, Latinos/Hispanics, and African Americans from ten independent studies participating in the Pharmacogenomics in Childhood Asthma (PiCA) Consortium [43] when assessing asthma exacerbations despite ICS use as an outcome. At genomic region level, a total of 5919 variants within 100 kilobases (kb) upstream and downstream from *ROBO2* were assessed in Europeans. From these, eleven SNPs were significantly associated with asthma exacerbations despite ICS use after accounting for the 164 independent variants located within this region (Bonferroni-like correction significance threshold of *p* ≤ 3.04 × 10^−4^). The SNP rs72891545 was the most significant association with ICS response using asthma exacerbations as the outcome (OR for A allele: 4.79, 95% CI: 2.36–9.73, *p* = 1.44 × 10^−5^) (Table S2, Figure S2). A total of 6453 variants within a +/−100 kb window from *ROBO2* were evaluated in admixed populations. However, no significant associations with asthma exacerbations despite ICS were found after applying a Bonferroni-like correction (*p* ≤ 1.22 × 10^−4^ for 411 independent variants).

### 2.4. Sensitivity Analyses Accounting for Asthma Severity

The association of the SNP rs1166980 with a binary variable of the change in FEV_1_ after ICS treatment remained statistically significant after also including basal FEV_1_ measurements as a covariate in the regression models (OR for G allele: 7.21, 95% CI: 3.15–16.50, *p* = 2.95 × 10^−6^). Similar results were found when a quantitative outcome of the change in FEV_1_ was tested in association (β for G allele: −5.58, 95% CI: −8.72–−2.44, *p* = 6.42 × 10^−4^).

Further sensitivity analyses were performed in 2282 individuals from six of the eight studies from PiCA from European descent populations with classification of asthma severity based on treatment steps [44]. Specifically, asthma severity was included as a covariate in the regression models for the variant rs72891545. No major differences were found (OR for A allele: 2.66, 95% CI: 1.58–4.45, *p* = 1.71 × 10^−3^) compared to the original association models performed for the subset of patients with available information about treatment steps (OR for A allele: 3.66, 95% CI: 2.08–6.43, *p* = 1.32 × 10^−4^).

### 2.5. In Silico Evaluation of the Variants Associated with Different Definitions of ICS Response

According to publicly available functional evidence from the Encyclopedia of DNA Elements (ENCODE) [45], the *ROBO2* intronic variant, rs1166980, associated with the change in FEV_1_ in SLOVENIA, could be involved in the regulation of the gene expression, since this nucleotide change is related to the modification of the binding site for different transcription factors (e.g., CTCF, Sox, and p300) [45]. Moreover, rs72891545, the most significant association signal with asthma exacerbations, has also been predicted to involve the modification of several transcription factor binding sites (ELF1, Myc, Sp4, YY1, Zfx). Although no previous evidence of significant expression quantitative trait loci (eQTL) or methylation quantitative trait loci (meQTL) was found for any of these variants [45], both have been significantly associated as protein quantitative trait loci (pQTL) (*p* ≤ 0.01) [46,47]. Specifically, rs1166980 has been associated with the expression of 38 different proteins in plasma (Table S3) [48,49,50], including some involved in the immune response (e.g., PI3, COLEC11, and TNFRSF21), inflammatory processes (HPGD), and drug metabolism (TBXAS1). Moreover, it has been evidenced to be associated with protein expression levels of PMEPA1, a negative regulator of the activity of transforming growth factor β (TGF-β), which has been widely proposed to play a key role in allergy and asthma pathophysiology [51]. On the other hand, rs72891545 had been previously associated with the expression levels of 19 proteins [49] implicated in different functions, including some related to asthma pathophysiology, such as the maintenance of the innate immunity (ERAP2, FCN1, and SIGLEC9), collagen deposition (COL8A1), and the TGF-β signaling pathway (MSTN) (Table S4). Additionally, several proteins, whose expression could be regulated by these variants, had been previously associated with asthma-related traits and/or allergic diseases (e.g., OLFML3, PCSK3, ZNF180, GALNT16, and KLK14) (Tables S3 and S4) [52].

### 2.6. Validation of Previous Associations with ICS Response

Among the 26 SNPs previously associated with ICS response through GWAS approaches in studies independent from the PiCA consortium (Table S5), two variants were found to be nominally associated with the binary outcome related to the change in FEV_1_ after 6 weeks of ICS treatment in the SLOVENIA study: rs2395672 at *CMTR1* (OR for G allele: 1.78, 95% CI: 1.03–3.05, *p* = 0.037) and rs3827907 at *EDDM3B* (OR for C allele: 0.52, 95% CI: 0.32–0.84, *p* = 7.40 × 10^−3^) (Table S6). However, these did not remain significant after adjusting for the total number of variants assessed (*p* ≤ 1.92 × 10^−3^). At the genomic-region level, a total of 33,617 variants located within a 100 kb window from genes previously associated with ICS response were assessed. This resulted in evidence of suggestive replication for two variants located in the intergenic region of *PDE10A* and *T* after Bonferroni-like correction of the significance threshold within each genomic region: rs9365939 (OR for G allele: 0.41, 95% CI: 0.26–0.65, *p* = 1.92 × 10^−4^) and rs2118353 (OR for T allele: 0.41, 95% CI: 0.26–0.65, *p* = 1.92 × 10^−4^) (Table S7). However, these associations would not be considered significant after correcting for the total number of independent SNPs tested across all the genomic regions (*p* ≤ 2.89 × 10^−5^ for 1728 independent variants).

## 3. Discussion

This is a pilot study describing the results of one of the few GWASs of the response to ICS in asthma carried out in ICS-naïve patients to date. A variant located in the *ROBO2* gene was the most significantly associated with a binary variable of ICS responsiveness based on FEV_1_ change after 6 weeks of ICS treatment. Consistent with this finding, the same result was also found with the quantitative variable of this change, although no evidence of replication with the same outcome was found in an independent study considering a wider timeframe. The association of *ROBO2* was validated at genomic-region level, by analyzing asthma exacerbations despite ICS use in Europeans. However, no evidence of replication was found in Latinos/Hispanics and African Americans. Moreover, the effects of the variants at *ROBO2* might be driven by the response to asthma treatment rather than disease severity since significance remained after including covariates as proxies of asthma severity.

*ROBO2* encodes one of the members of the roundabout guidance receptor’s family, which are immunoglobulins highly conserved across species. Four ROBO proteins have been identified in humans [53]. These are transmembrane receptors binding Slit guidance ligands (SLIT) [53,54,55] with functions initially linked to the development of the nervous system, including the modulation of axon guidance and cell migration [53,54,55]. However, they have also been demonstrated to be involved in different processes, including some that are part of the asthma pathophysiology. Specifically, ROBO/SLIT has been related to cell adhesion, migration, growth, and survival [56], in addition to the morphogenesis of normal and aberrant pulmonary tissues [56]. There is also some evidence suggesting the implication of the ROBO signaling pathway in the regulation of innate immunity [57,58]. Previous studies have related the increased production of chemoattractants with the inhibition of the ROBO expression, promoting the migration of immune cells from the blood circulatory system to the pulmonary tissue and airway remodeling processes, decreasing the number of alveoli [59]. Specifically, ROBO2 is involved in the signal transduction of SLIT2 [55], which has been shown to have an important function in pulmonary diseases [55,58,60]. SLIT2 is implicated in the regulation of chemotaxis and migration of several types of immune cells into the lung (e.g., leukocytes, T lymphocytes, dendritic cells, macrophages, and neutrophils), preventing inflammatory processes [57,61,62]. Nonetheless, members of ROBO/SLIT have also been found to be involved in the eosinophil chemotaxis induced by eotaxin, enhancing allergic airway inflammation [63]. Although little is known about its role in allergic processes outside the respiratory system, these pieces of evidence suggest its potential implication in different types of allergic responses. In addition, SLIT2 has been found to inhibit the migration of monocytes from the systemic circulation and their differentiation into fibrocytes in the lung, proliferation of fibroblasts, and collagen production through TGF-β activity [64]. SLIT2 and ROBO2 have been evidenced to prevent fibrotic processes in several diseases, including pulmonary fibrosis [64]. Moreover, Lin et al_._ detected decreased levels of ROBO2 and SLIT2 in chronic obstructive pulmonary disease (COPD) patients [58], a disease with underlying mechanisms shared with asthma [65,66].

These findings suggest that SLIT ligands could be involved in anti-inflammatory and antifibrotic processes. Thus, ROBO proteins could also be involved in processes occurring later in life with important implications in pulmonary disorders [57,64]. Interestingly, Ning et al. found that ROBO2/SLIT2 could be involved in the migration of rat airway smooth muscle (ASM) cells within the airway wall [67], which has been proposed to be a key feature of the structural changes taking place in asthma pathophysiology [68]. The migration of ASM induced by platelet-derived growth factor was detected to be inhibited by the administration of SLIT2 [67]. Furthermore, *ROBO2* has been evidenced to be an important factor in triggering airways constriction in asthma and COPD [69]. The expression levels of *ROBO2* and *SLIT2* have been negatively correlated with COPD progression [58]. Specifically, it has been hypothesized that the downregulation of both genes could activate Cdc42 and Rac2 GTPases, promoting the migration of neutrophils and T lymphocytes into the lung, triggering inflammation [58], a mechanism that could also take place in asthma.

Additionally, *ROBO2* has been suggestively associated with post-bronchodilator spirometric measures in African Americans. Specifically, three intronic *ROBO2* variants were associated with FEV_1_ and the ratio between FEV_1_ and the forced vital capacity (FVC) measured after the administration of short-acting β2 agonists (SABA) [70]. Furthermore, this gene was identified to be a shared genetic factor for asthma susceptibility among European, African American, and Latino/Hispanic populations [71]. Ding et al. also suggested that *ROBO2* may be part of biological networks related to inflammatory diseases and disorders of the immune system [71]. Altogether, this evidence suggests that *ROBO2* could play an important role in asthma phenotypes, including the response to ICS in asthma.

The findings of this study suggest the potential implication of *ROBO2* in the response to asthma treatment with ICS specifically in European populations, with a lack of evidence of replication in Latinos/Hispanics and African Americans. This could be explained by the fact that Latinos/Hispanics and African Americans widely differ from homogenous populations in terms of linkage disequilibrium patterns, allele frequency, gene–gene, and gene–environment interactions as a result of the recent admixture processes as the origin of these populations [72]. In fact, previous studies have suggested the potential existence of ancestry-specific genetic variation in the response to this medication [38,39].

As part of this study, we also assessed the replication of SNPs and genes that have previously been implicated in ICS response, providing evidence of an association of the intergenic region of the *PDE10A* and *T* genes with FEV_1_ change after ICS treatment. However, this association was demonstrated for different SNPs from those described in the study reporting the association of this genomic region with ICS response [28]. Interestingly, evidence of replication had also been found for this locus with ICS response measured as the occurrence or absence of asthma exacerbations in European populations [39]. These findings suggest that *PDE10A*-*T* could play an important role in ICS response in asthma patients, at least those of European descent. Furthermore, the association of this locus with ICS response was first identified in children and adults, which has been now replicated in children and young adults, suggesting the potential existence of some genetic factors shared between childhood and adulthood asthma. However, further investigation is needed to be able to better understand the genetic contribution to asthma treatment response in different age groups.

The preliminary results of this study demonstrate the importance of omics approaches to provide insights into asthma-related traits. Nonetheless, further investigation of the genetic factors underlying ICS response in combination with the assessment of the molecular modification of the individual’s genetic material in response to environmental exposures is crucial to better understand the mechanisms underlying ICS responsiveness in asthma patients [73,74].

This study has several limitations that need to be acknowledged. First, the sample size of the discovery phase was limited, which could cause only one variant to be suggestively associated with ICS responsiveness, with a lack of genome-wide significant associations. Nonetheless, the fact that FEV_1_ was measured in ICS-naïve patients with asthma, an approach that is rare among studies, explains part of the difficulty in achieving a larger sample size. Second, information related to spirometry recordings before and after a short period of ICS treatment was available only in one independent study, and no evidence of replication using the same measurement was found, which could be explained by the reduced number of individuals included in the analyses and the different timeframe considered. However, the association of *ROBO2* with ICS response among asthma patients was also found when evaluating the association with asthma exacerbations despite ICS treatment. Third, different definitions of asthma exacerbations were used based on retrospective information from European and admixed asthmatic children treated with ICS, which could not be fully informative about the response to asthma treatment. Fourth, information about the specific type of ICS used and the doses administered, or indices of treatment adherence were not available for any of the studies evaluated. Fifth, covariates related to the type of asthma, exposure to environmental triggers, or the presence of comorbidities were not included in the analyses given the lack of such information.

In conclusion, this study suggests the association with a variant in *ROBO2* and the change in FEV_1_ after ICS treatment in European children and young adults with asthma. This association was validated using asthma exacerbations despite ICS use as an alternative outcome in independent European and admixed populations. Taken together with the biologically plausible role of *ROBO2* in pulmonary and immune functions, *ROBO2* potentially represents a novel locus determining the response to ICS in patients with asthma. Larger studies coupled with functional evaluation are required to fully understand the role of *ROBO2* in responsiveness to ICS in patients with asthma.

## 4. Materials and Methods

### 4.1. Study Population Analyzed in the Discovery Phase

Patients with mild-to-moderate persistent asthma from the SLOVENIA study were included in the GWAS of change in lung function after ICS treatment. Children and young adults (5–18 years old) of Slovenian origin were included in this study. Co-existence of other chronic inflammatory disorders, except for asthma and atopic diseases, was considered as an exclusion criterion [38,41].

A subset of patients with reports of at least one use of any type of ICS and/or combination with long-acting β2 agonists (LABA) in the 12 months preceding the study enrolment was analyzed. Availability of genome-wide genotypes, data on the change in FEV_1_ after 6 weeks of ICS therapy, and information regarding asthma exacerbations were considered as inclusion criteria in the GWAS analyses. FEV_1_, expressed as the percentage of the predicted value based on sex, age, and height of the patients, was measured before the beginning of ICS treatment (when the patients were ICS-naïve) and 6 weeks after the start of the treatment using a Vitalograph 2150 spirometer (Compact, Buckingham, UK), according to the standard guidelines [41,75]. On the basis of these measurements, the percent change in FEV_1_ (ΔFEV_1_) was calculated as (post-FEV1–pre-FEV1)/(pre-FEV1) × 100. Based on a threshold of 8% FEV_1_ improvement, which has been shown to be a good predictor of asthma treatment response in children [38,76], participants were classified as ICS responders (ΔFEV_1_ ≥ 8%) or non-responders (ΔFEV_1_ < 8%).

### 4.2. Genotyping and Imputation of Genetic Variants in SLOVENIA

The SLOVENIA samples were genotyped using the Illumina Global Screening Array-24 v1.0 BeadChip (Illumina Inc., San Diego, CA, USA). Quality control (QC) analyses were carried out with PLINK 1.9 [77,78] and genetic variants across the whole genome were imputed following the procedures described elsewhere [38,39].

### 4.3. Association Testing with the Change in FEV_1_ Defined as a Binary Variable

The association of genetic variants with the binary variable of ICS response was tested using logistic regression models with the binary Wald test implemented in EPACTS 3.2.6 [79], including age and sex as covariates. Association analyses were also adjusted by the first two principal components (PCs) of genetic ancestry estimated by means of EIGENSOFT [80]. This model was selected since it showed the best fit with the expected values in terms of significance, assuming no association as null hypothesis attending to λ_GC_ values, estimated through the R package *gap* [81], and quantile–quantile plots.

Results were filtered to retain common SNPs (MAF ≥ 1%), and imputation quality Rsq ≥ 0.3 and variants that reached a significance threshold of *p*-value ≤ 5 × 10^−6^ were deemed suggestively associated and followed up for replication in additional studies. This threshold was set arbitrarily, following what has been commonly adopted by previous GWAS [38,39].

### 4.4. Association with the Quantitative Change in FEV_1_ after ICS Treatment

SNPs suggestively associated with the binary outcome related to the change in FEV_1_ after 6 weeks of ICS treatment were assessed in the same group of asthma patients from the SLOVENIA study, but evaluating the association with the quantitative form of this outcome. Linear regression models were performed through linear Wald tests in EPACTS 3.2.6 [79], adjusted by the same aforementioned covariates.

Validation was also attempted with the change in FEV_1_ after a short period under ICS therapy in an independent study of European ancestry. Asthma patients with reported use of ICS in the previous 12 months from the CAMP study were included in the analyses (Table S1). ICS responders and non-responders were classified based on the change in FEV_1_ after 2 months of ICS use using the same criteria considered in the discovery phase. Genetic variants were imputed using phase 3 of the 1000 Genomes Project (1KGP) [82] through the Michigan Imputation Server [83]. Further information is described in the Supplementary Material. Binary and quantitative variables of the ICS response, measured as the change in FEV_1_, were tested in association through logistic and linear regressions using PLINK 1.9 [77,78], respectively. Validation of the association was considered for nominally significant variants (*p*-value ≤ 0.05) with the same direction of the association effect as in the discovery phase.

### 4.5. Replication of Results Analyzing the Association with Asthma Exacerbations despite ICS Use in Additional Studies

The genetic markers found to be associated with the change in FEV_1_ after ICS treatment were attempted for validation with the absence or presence of asthma exacerbations despite the use of ICS. This was done in ten independent studies included in the PiCA consortium [43]. Association testing was undertaken in asthma patients (2–25 years old) treated with ICS in the previous year, separately performed in two groups of studies on the basis of their ancestry.

On one hand, eight independent European studies were analyzed: BREATHE; Effectiveness and Safety of Treatment with Asthma Therapy in Children (ESTATe); the follow-up stage of the Multicentre Asthma Genetics in Childhood Study (followMAGICS); Genetics of the Scottish Health Research Register (GoSHARE); the Pharmacogenetics of Asthma medication in Children: Medication with Anti-Inflammatory effects (PACMAN); the Pediatric Asthma Gene Environment Study (PAGES); the Pharmacogenetics of Adrenal Suppression with Inhaled Steroids (PASS) study; SLOVENIA. Additionally, two recently admixed populations with African ancestry were also included in association analyses: Latinos/Hispanics and African Americans from the Genes–Environment and Admixture in Latino Americans (GALA II) study, and African Americans included in the Study of African Americans, Asthma, Genes, and Environments (SAGE), respectively.

Severe asthma exacerbations were defined as the need for emergency care, hospitalizations, or systemically administered corticosteroids because of asthma in the previous 6 or 12 months depending on the study. Alternatively, moderated exacerbations evidenced by unscheduled general practitioner or respiratory system specialist visits and school absences were considered for BREATHE–PAGES, BREATHE, and followMAGICS given the lack of information. ICS use was defined using the same criteria described for participants in the SLOVENIA study. Further description of the characteristics of the study populations, genotyping, imputation, and association analyses are available in the Supplementary Material and elsewhere [38,39,42].

Association with the presence/absence of asthma exacerbations despite ICS use was assessed in each study using the same methodology described in previous publications [38,39,42]. The association of the SNPs identified in the discovery phase was separately evaluated in each ancestry group of studies. Replication was carried out at the SNP level, but also genomic regions were considered, including variants located within a 100 kb window upstream and downstream from the limits of the genes where the variants were located. Only common SNPs with MAF ≥ 1% and Rsq ≥ 0.3 shared among the populations included in each group were included. Replication results were considered significant for those SNPs that reached the Bonferroni-corrected significance threshold, estimated as α = 0.05/number of independent signals within each genomic region, an approach considered to provide the closest approximation to permutation-based methods [84]. For this, independent variants were separately estimated for Europeans and non-Europeans through empirical autocorrelations based on the –log_10_ *p*-value of each SNP analyzed using the R package coda [77,78,85].

### 4.6. Sensitivity Analyses of Asthma Treatment Response

Sensitivity analyses were carried out for the variants identified to ascertain whether the association effect detected was driven by disease severity rather than a response to asthma medications. First, regression analyses evaluating the association with the binary variable of the change in FEV_1_ after ICS therapy were also adjusted by the FEV_1_ measured at the beginning of ICS therapy. On the other hand, a modified classification into treatment steps [44] as a proxy of asthma severity was included as a covariate. Nonetheless, this could not be carried out in SLOVENIA, since this study had incomplete information about the different medications included in the definition of treatment steps. Therefore, sensitivity analyses accounting for treatment step classification assessing the association with asthma exacerbations despite ICS use. The same methodology previously described was applied [39,42].

### 4.7. In Silico Functional Evaluation of Variants Associated with ICS Response

The potential functional implications of the variants suggestively associated with the change in FEV_1_ after 6 weeks of ICS treatment in SLOVENIA and those with evidence of replication with asthma exacerbations in ICS users were assessed using several publicly available databases. The potential role as eQTL, DNase hypersensitivity sites, and histone marks was assessed using HaploReg v4.1 [86], based on the functional evidence from ENCODE [45]. Significant evidence as pQTL or meQTL previously reported by publicly available studies was also explored using the PhenoScanner v2 tool [46,47].

### 4.8. Validation of Previous Associations with ICS Response

Previous GWASs, apart from those carried out as part of the PiCA consortium, had identified a total of 26 SNPs near or within 15 genes associated with ICS response in several populations (Table S5). Validation of these associations was attempted at SNP level using the results of the GWAS of the binary variable of the change in FEV_1_ after ICS treatment performed in the SLOVENIA study. Evidence of replication was considered for significant variants at the nominal level (*p* ≤ 0.05) with the same direction of the association effect as in the discovery phase. Replication was also evaluated at the genomic-region level, including a 100 kb window from the limits of the genes of ICS response previously identified. A Bonferroni-like correction was applied accounting for the number of independent variants analyzed within each genomic region. To avoid being too stringent and conservative, evidence of replication was considered for those association signals reaching the Bonferroni-corrected significance threshold estimated as α = 0.05/number of independent variants [38,39] instead of considering all the variants within the region, which could be correlated by LD.

## Figures and Tables

**Table 1:** Clinical and demographic characteristics of the asthma patients from the SLOVENIA study included in the GWAS of change in FEV_1_ after ICS treatment.

	Total	ICS Non- Responders ^a^	ICS Responders ^b^	*p*-Value
Sample size	166	94	72	-
Gender, *n* (% male)	98 (59.0)	59 (62.8)	39 (54.2)	0.264 ^e^
Mean age ± SD (years)	10.9 ± 3.4	10.7 ± 3.2	11.2 ± 3.5	0.461 ^f^
Lung function				
Mean basal FEV_1_ ± SD (%) ^c^	87.1 ± 14.8	91.3 ± 12.7	81.6 ± 15.5	<0.001 ^f^
Mean post-treatment FEV_1_ ± SD (%) ^d^	93.7 ± 14.4	90.1 ± 13.6	98.5 ± 14.2	<0.001 ^f^
Mean ΔFEV_1_ ± SD (%)	6.7 ± 12.1	−1.2 ± 7.8	16.9 ± 8.7	<0.001 ^f^

^a^ Asthma patients with ΔFEV_1_ < 8% after 6 weeks of ICS treatment; ^b^ Asthma patients with ΔFEV_1_ ≥ 8% after 6 weeks of ICS treatment; ^c^ FEV_1_ measured at the beginning of ICS treatment; ^d^ FEV_1_ measured after 6 weeks of ICS treatment; ^e^ Pearson χ2 test (df = 1; α = 0.05); ^f^ Mann–Whitney U test. FEV_1_: forced expiratory volume in one second; ΔFEV_1_: change in FEV_1_ after 6 weeks of ICS treatment; SD: standard deviation; NA: not available.

## Data Availability

The data presented in this study are available on request from the corresponding author.

## Supplementary Materials

The following are available online at https://www.mdpi.com/article/10.3390/jpm11080733/s1, Supplementary methods, Figure S1: Quantile-quantile plot of association results of ICS response measured as the binary outcome related to the change in FEV_1_ after ICS treatment, Figure S2: Regional plot of association results with asthma exacerbations despite ICS use in European children and young adults, Table S1: Clinical and demographic characteristics of the asthma patients treated with ICS from CAMP included in the evaluation of the association with the change in FEV_1_ after ICS therapy, Table S2: Genomic-region replication of *ROBO2* with asthma exacerbations despite ICS use in European populations. Evidence for significant variants after Bonferroni-like correction, Table S3: Proteins with expression levels in plasma associated with rs1166980, Table S4: Proteins with expression levels affected by the SNP rs72891545, Table S5: Genes associated with ICS response by genome-wide association studies published to date, Table S6: Results of SNP-level replication of previous associations of ICS response in the results of the GWAS of the change in FEV_1_ after ICS treatment performed in the SLOVENIA study, Table S7: Genomic-region replication of previous associations of ICS response. Evidence of association with the change in FEV_1_ after ICS treatment in asthma patients.
