# Supplementary material for: Identification of ROBO2 as a Potential Locus Associated with Inhaled Corticosteroid Response in Childhood Asthma
Source: J Pers Med. 2021 Jul 28;11(8):733. doi: 10.3390/jpm11080733 (PMC8399629; doi:10.3390/jpm11080733)
Supplement: Supplementary file 1 [file jpm-11-00733-s001.zip › SM_Authors_correction.pdf]

Journal of
Personalized Medicine

MDPI

1. Supplementary methods

1.1. Design and characteristics of the independent study included in the assessment of the association with the change in FEV1 after ICS use

CAMP (n = 175)

Childhood Asthma Management Program (CAMP) is a longitudinal study initially designed as a clinical trial for the evaluation of the multiple side-effects of the long-term use of corticosteroids. Childhood patients (5-12 years old) with a clinical diagnosis of chronic asthma were included, whereas those subjects with severe asthma and other respiratory conditions were excluded [S1–3].

1.2. Design and characteristics of the studies included in association analyses with asthma exacerbations despite ICS use

European populations

PACMAN (n = 654)

The Pharmacogenetics of Asthma medication in Children: Medication with Anti-inflammatory effects (PACMAN) study is an observational cohort including children (4-12 years old) with self-reported use of any asthma medications. Recruitment was carried out through records of community pharmacies in the Netherlands [S4].

PAGES (n = 437)

The Paediatric Asthma Gene Environment Study (PAGES) is a cross-sectional study that includes asthma patients (2-16 years old) with a pediatrician's diagnosis, recruited at secondary care clinics at different centers across the United Kingdom: Aberdeen, Edinburg, Glasgow, Kilmarnock, and Brighton. Clinical assessment through questionnaires about dietary and quality of life was complimented, and saliva samples were collected. The coexistence of any respiratory diseases or significant health problems was considered as exclusion criteria [S5, S6].

BREATHE (n = 288)

The BREATHE study recruited participants aged 3 to 22 years old with a physician's diagnosis of asthma. Participants were recruited at primary and secondary care centers from the United Kingdom [S7–9]. From the BREATHE samples included, genotypes from 182 patients had been obtained using the Illumina Infinium CoreExome-24 BeadChip (Illumina) array, whereas 103 samples were genotyped using the Axiom™ Precision Medicine Research Array (Affymetrix Inc.) [S6]. Quality control procedures were applied as described in Hernandez-Pacheco *et al.* [S10]. Association analyses were performed for the latter together with PAGES samples due to similarities of study design and sample characteristics, denoted as BREATHE-PAGES [S6].

GoSHARE (n = 472)

The Genetic of the Scottish Health Research Register (GoSHARE) recruited children and young adults (3-18 years old) in Tayside (Scotland) through complete electronic medical records (EMR) available at databases from the National Health Service [S11].

PASS (n = 402)

The Pharmacogenetics of Adrenal Suppression with Inhaled Steroids study (PASS) includes children and young adults (5-18 years old) from the United Kingdom. Participants had a physician's diagnosis of asthma and were treated with inhaled corticosteroids (ICS) under medical supervision. Clinical concern about adrenal suppression was also considered as an inclusion criterion since this study was initially conceived to explore the effects of corticosteroids on adrenal suppression [S12, S13].

SLOVENIA (n = 182)

The SLOVENIA study recruited patients (5-18 years old) with a physician diagnosis of mild to moderate persistent asthma and hospital records at tertiary health centers in Slovenia. Part of the participants was regularly treated with ICS under medical supervision. The coexistence of other chronic inflammatory diseases was considered as an exclusion criterion [S14].

followMAGICS (n = 147)

Participants with persistent asthma symptoms from the follow-up phase of the Multicentre Asthma Genetics in Childhood Study (followMAGICS) were aged from 7 to 25 years old. Children with a physician's diagnosis of asthma were recruited at secondary and tertiary centers from Germany and Austria [S15–18].

ESTATe (n = 102)

Children and young adults (4-19 years old) with a physician's diagnosis of asthma were included in the case-control Effectiveness and Safety of Treatment with Asthma Therapy in children (ESTATe) study. Patients using any asthma controller medication were recruited at primary care units from the Netherlands based on electronic medical records [S10].

Admixed populations

GALA II (n = 854)

Genes-Environment and Admixture in Latino Americans (GALA II) is a case-control study including asthma patients (8-21 years old) with a physician's diagnosis, active symptoms, and reported use of any asthma medications recruited in the United States and Puerto Rico. Hispanic/Latino origin based on four grandparents belonging to that ancestry group was used as an inclusion criterion [S19].

SAGE (n = 493)

The Study of African Americans, Asthma, Genes, and Environments (SAGE) recruited asthma patients following the same protocols used in GALA II. Individuals with four grandparents of African American ancestry were recruited in several centers across the United States [S19, S20].

Supplementary references

S1. Design and Implementation of a Patient Education Center for the Childhood Asthma Management Program. Childhood Asthma Management Program Research Group. *Ann Allergy Asthma Immunol* **1998**, *81* (6), 571–581.

S2. The Childhood Asthma Management Program (CAMP): Design, Rationale, and Methods. Childhood Asthma Management Program Research Group. *Control Clin Trials* **1999**, *20* (1), 91–120.

S3. Zeiger, R. S.; Dawson, C.; Weiss, S. Relationships between Duration of Asthma and Asthma Severity among Children in the Childhood Asthma Management Program (CAMP). *J Allergy Clin Immunol* **1999**, *103* (3 Pt 1), 376–387.

S4. Koster, E. S.; Raaijmakers, J. A.; Koppelman, G. H.; Postma, D. S.; van der Ent, C. K.; Koenderman, L.; Bracke, M.; Maitland-van der Zee, A. H. Pharmacogenetics of Anti-Inflammatory Treatment in Children with Asthma: Rationale and Design of the PACMAN Cohort. *Pharmacogenomics* **2009**, *10* (8), 1351–1361.

S5. Turner, S. W.; Ayres, J. G.; Macfarlane, T. V; Mehta, A.; Mehta, G.; Palmer, C. N.; Cunningham, S.; Adams, T.; Aniruddhan, K.; Bell, C.; Corrigan, D.; Cunningham, J.; Duncan, A.; Hunt, G.; Leece, R.; MacFadyen, U.; McCormick, J.; McLeish, S.; Mitra, A.; Miller, D.; Waxman, E.; Webb, A.; Wojcik, S.; Mukhopadhyay, S.; Macgregor, D. A Methodology to Establish a Database to Study Gene

Environment Interactions for Childhood Asthma. *BMC Med Res Methodol* **2010**, *10*, 107.

S6. Hernandez-Pacheco, N.; Vijverberg, S. J.; Herrera-Luis, E.; Li, J.; Sio, Y. Y.; Granell, R.; Corrales, A.; Maroteau, C.; Lethem, R.; Perez-Garcia, J.; Farzan, N.; Repnik, K.; Gorenjak, M.; Soares, P.; Karimi, L.; Schieck, M.; Perez-Mendez, L.; Berce, V.; Tavendale, R.; Eng, C.; Sardon, O.; Kull, I.; Mukhopadhyay, S.; Pirmohamed, M.; Verhamme, K. M.; Burchard, E. G.; Kabesch, M.; Hawcutt, D. B.; Melen, E.; Potocnik, U.; Chew, F. T.; Tantisira, K. G.; Turner, S.; Palmer, C. N.; Flores, C.; Pino-Yanes, M.; Maitland-van der Zee, A. H. Genome-Wide Association Study of Asthma Exacerbations despite Inhaled Corticosteroids Use. *Eur Respir J* **2020**, *57* (5), 2003388.

S7. Palmer, C. N.; Doney, A. S.; Lee, S. P.; Murrie, I.; Ismail, T.; Macgregor, D. F.; Mukhopadhyay, S. Glutathione S-Transferase M1 and P1 Genotype, Passive Smoking, and Peak Expiratory Flow in Asthma. *Pediatrics* **2006**, *118* (2), 710–716.

S8. Palmer, C. N.; Lipworth, B. J.; Lee, S.; Ismail, T.; Macgregor, D. F.; Mukhopadhyay, S. Arginine-16 Beta2 Adrenoceptor Genotype Predisposes to Exacerbations in Young Asthmatics Taking Regular Salmeterol. *Thorax* **2006**, *61* (11), 940–944.

S9. Tavendale, R.; Macgregor, D. F.; Mukhopadhyay, S.; Palmer, C. N. A Polymorphism Controlling *ORMDL3* Expression Is Associated with Asthma That Is Poorly Controlled by Current Medications. *J Allergy Clin Immunol* **2008**, *121* (4), 860–863.

S10. Hernandez-Pacheco, N.; Farzan, N.; Francis, B.; Karimi, L.; Repnik, K.; Vijverberg, S. J.; Soares, P.; Schieck, M.; Gorenjak, M.; Forno, E.; Eng, C.; Oh, S. S.; Perez-Mendez, L.; Berce, V.; Tavendale, R.; Samedy, L. A.; Hunstman, S.; Hu, D.; Meade, K.; Farber, H. J.; Avila, P. C.; Serebrisky, D.; Thyne, S. M.; Brigino-Buenaventura, E.; Rodriguez-Cintron, W.; Sen, S.; Kumar, R.; Lenoir, M.; Rodriguez-Santana, J. R.; Celedon, J. C.; Mukhopadhyay, S.; Potocnik, U.; Pirmohamed, M.; Verhamme, K. M.; Kabesch, M.; Palmer, C. N. A.; Hawcutt, D. B.; Flores, C.; Maitland-van der Zee, A. H.; Burchard, E. G.; Pino-Yanes, M. Genome-Wide Association Study of Inhaled Corticosteroid Response in Admixed Children with Asthma. *Clin Exp Allergy* **2019**, *49* (6), 789–798.

S11. McKinstry, B.; Sullivan, F. M.; Vasishta, S.; Armstrong, R.; Hanley, J.; Haughney, J.; Philip, S.; Smith, B. H.; Wood, A.; Palmer, C. N. Cohort Profile: The Scottish Research Register SHARE. A Register of People Interested in Research Participation Linked to NHS Data Sets. *BMJ Open* **2017**, *7* (2), e013351.

S12. Hawcutt, D. B.; Jorgensen, A. L.; Wallin, N.; Thompson, B.; Peak, M.; Lacy, D.; Newland, P.; Didi, M.; Couriel, J.; Blair, J.; Pirmohamed, M.; Smyth, R. L. Adrenal Responses to a Low-Dose Short Synacthen Test in Children with Asthma. *Clin Endocrinol* **2015**, *82* (5), 648–656.

S13. Hawcutt, D. B.; Francis, B.; Carr, D. F.; Jorgensen, A. L.; Yin, P.; Wallin, N.; O'Hara, N.; Zhang, E. J.; Bloch, K. M.; Ganguli, A.; Thompson, B.; McEvoy, L.; Peak, M.; Crawford, A. A.; Walker, B. R.; Blair, J. C.; Couriel, J.; Smyth, R. L.; Pirmohamed, M. Susceptibility to Corticosteroid-Induced Adrenal Suppression: A Genome-Wide Association Study. *Lancet Respir Med* **2018**, *6*(6), 442-450.

S14. Berce, V.; Kozmus, C. E.; Potocnik, U. Association among ORMDL3 Gene Expression, 17q21 Polymorphism and Response to Treatment with Inhaled Corticosteroids in Children with Asthma. *Pharmacogenomics J* **2013**, *13* (6), 523–529.

S15. Moffatt, M. F.; Kabesch, M.; Liang, L.; Dixon, A. L.; Strachan, D.; Heath, S.; Depner, M.; von Berg, A.; Bufe, A.; Rietschel, E.; Heinzmann, A.; Simma, B.; Frischer, T.; Willis-Owen, S. A.; Wong, K. C.; Illig, T.; Vogelberg, C.; Weiland, S. K.; von Mutius, E.; Abecasis, G. R.; Farrall, M.; Gut, I. G.; Lathrop, G. M.; Cookson, W. O. Genetic Variants Regulating *ORMDL3* Expression Contribute to the Risk of Childhood Asthma. *Nature* **2007**, *448* (7152), 470–473.

S16. Pandey, R. C.; Michel, S.; Schieck, M.; Binia, A.; Liang, L.; Klopp, N.; Franke, A.; von Berg, A.; Bufe, A.; Rietschel, E.; Heinzmann, A.; Laub, O.; Simma, B.; Frischer, T.; Genuneit, J.; Illig, T.; Kabesch, M. Polymorphisms in Extracellular Signal-Regulated Kinase Family Influence Genetic Susceptibility to Asthma. *J Allergy Clin Immunol* **2013**, *131* (4), 1245–1247.

S17. Schieck, M.; Michel, S.; Suttner, K.; Illig, T.; Zeilinger, S.; Franke, A.; Vogelberg, C.; von Berg, A.; Bufe, A.; Heinzmann, A.; Laub, O.; Rietschel, E.; Simma, B.; Frischer, T.; Genuneit, J.; Kerzel, S.; Kabesch, M. Genetic Variation in TH17 Pathway Genes, Childhood Asthma, and Total Serum IgE Levels. *J Allergy Clin Immunol* **2014**, *133* (3), 888–891.

S18. Nieuwenhuis, M. A.; Siedlinski, M.; van den Berge, M.; Granell, R.; Li, X.; Niens, M.; van der Vlies, P.; Altmuller, J.; Nurnberg, P.; Kerkhof, M.; van Schayck, O. C.; Riemersma, R. A.; van der Molen, T.; de Monchy, J. G.; Bosse, Y.; Sandford, A.; Bruijnzeel-Koomen, C. A.; Gerth van Wijk, R.; Ten Hacken, N. H.; Timens, W.; Boezen, H. M.; Henderson, J.; Kabesch, M.; Vonk, J. M.; Postma, D. S.; Koppelman, G. H. Combining Genomewide Association Study and Lung EQTL Analysis Provides Evidence for Novel Genes Associated with Asthma. *Allergy* **2016**, *71* (12), 1712–1720.

S19. Nishimura, K. K.; Galanter, J. M.; Roth, L. A.; Oh, S. S.; Thakur, N.; Nguyen, E. A.; Thyne, S.; Farber, H. J.; Serebrisky, D.; Kumar, R.; Brigino-Buenaventura, E.; Davis, A.; LeNoir, M. A.; Meade, K.; Rodriguez-Cintron, W.; Avila, P. C.; Borrell, L. N.; Bibbins-Domingo, K.; Rodriguez-Santana, J. R.; Sen, S.; Lurmann, F.; Balmes, J. R.; Burchard, E. G. Early-Life Air Pollution and Asthma Risk in Minority Children. The GALA II and SAGE II Studies. *Am J Respir Crit Care Med* **2013**, *188* (3), 309–318.

S20. Neophytou, A. M.; White, M. J.; Oh, S. S.; Thakur, N.; Galanter, J. M.; Nishimura, K. K.; Pino-Yanes, M.; Torgerson, D. G.; Gignoux, C. R.; Eng, C.; Nguyen, E. A.; Hu, D.; Mak, A. C.; Kumar, R.; Seibold, M. A.; Davis, A.; Farber, H. J.; Meade, K.; Avila, P. C.; Serebrisky, D.; Lenoir, M. A.; Brigino-Buenaventura, E.; Rodriguez-Cintron, W.; Bibbins-Domingo, K.; Thyne, S. M.; Williams, L. K.; Sen, S.; Gilliland, F. D.; Gauderman, W. J.; Rodriguez-Santana, J. R.; Lurmann, F.; Balmes, J. R.; Eisen, E. A.; Burchard, E. G. Air Pollution and Lung Function in Minority Youth with Asthma in the GALA II & SAGE II Studies. *Am J Respir Crit Care Med* **2016**, *193*(11), 1271-1280.

Table S1. Clinical and demographic characteristics of the asthma patients treated with ICS from the CAMP study included in the evaluation of the association with the changes in FEV_1 after ICS therapy.

	**Total**	**ICS non-responders** [a]	**ICS responders** [b]	***p*-value**
Sample size	173	93	80	-
Gender, n (% male)	98 (56.6)	52 (55.9)	46 (57.5)	0.960 [i]
Mean age ± SD (years)	8.9 ± 2.1	8.8 ± 1.9	9.0 ± 2.3	0.460 [j]
Lung function				
Mean basal FEV_1 ± SD (%) [c]	93.7 ± 14.8	98.7 ± 13.2	87.9 ± 14.5	$6.47e^{-6}$ [j]
Mean post-treatment FEV_1 ± SD (%) [d]	99.5 ± 14.8	99.9 ± 13.0	99.1 ± 16.8	0.990 [j]
Mean ΔFEV_1 ± SD (%)	5.8 ± 11.7	1.2 ± 3.8	11.2 ± 15.1	2.17×10^{-12} [j]
Asthma exacerbations in the last 12 months, n (%) [e]	80 (46.5)	42 (45.2)	38 (47.5)	0.820 [i]
ER visits, n (%) [f]	42 (24.3)	22 (23.7)	20 (25.0)	0.980 [i]
OCS use, n (%) [g]	64 (37.2)	29 (31.2)	35 (43.8)	1 [i]
Hospitalizations, n (%) [h]	13 (7.5)	7 (7.5)	6 (7.5)	0.780 [i]

[a] Asthma patients with $\Delta FEV_1 < 8\%$ after 2 months of ICS treatment; [b] Asthma patients with $\Delta FEV_1 \geq 8\%$ after 2 months of ICS treatment; [c] FEV_1 measured before the beginning of ICS therapy; [d] FEV_1 measured after 2 months of ICS treatment; [e] Defined as any emergency room visits, use of oral corticosteroids and/or hospitalizations because of asthma; [f] Proportion of patients with any exacerbations who sought emergency care due to asthma; [g] Proportion of patients with any exacerbations who needed the use oral corticosteroids because of asthma; [h] Proportion of patients with any exacerbations who needed to be hospitalized because of asthma; [i] Pearson $\chi 2$ test (df=1; α=0.05); [j] Mann-Whitney U test.

FEV_1: forced expiratory volume in one second; ΔFEV_1: change in FEV_1 after 2 months of ICS treatment; SD: standard deviation; NA: not available.

Table S2. Genomic-region replication of *ROBO2* with asthma exacerbations despite ICS use in European populations. Evidence for significant variants after Bonferroni-like correction.

SNP	Chr. [a]	Position [b]	E/NE	OR (95% CI) [c]	p-value
rs72891542	3	77183058	T/C	4.21 (2.12 – 8.37)	3.96×10^{-5}
rs72891545	3	77186033	A/G	4.79 (2.36 – 9.73)	**1.44×10^{-5}**
rs80109563	3	77189324	T/C	6.38 (2.60 – 15.65)	5.26×10^{-5}
rs77698848	3	77191168	A/G	6.06 (2.37 – 15.52)	1.72×10^{-4}
rs75844835	3	77192763	G/T	9.02 (3.04 – 26.79)	7.53×10^{-5}
rs75804244	3	77193590	A/G	6.47 (2.74 – 15.30)	2.11×10^{-5}
rs75336627	3	77197609	A/G	4.95 (2.18 – 11.27)	1.38×10^{-4}
rs77225325	3	77199127	A/C	4.95 (2.18 – 11.27)	1.38×10^{-4}
rs76099377	3	77199443	G/A	4.95 (2.18 – 11.27)	1.38×10^{-4}
rs7623806	3	77201032	C/T	4.95 (2.18 – 11.27)	1.38×10^{-4}
rs72891555	3	77205263	G/T	4.94 (2.09 – 11.71)	2.82×10^{-4}

[a] Chromosome; [b] Positions based on GRCh37/hg19 build; [c] Odds ratio for the effect alleles.

CI: Confidence Interval; E: Effect allele; ICS: inhaled corticosteroids; NA: not available; NE: Non-effect allele; SNP: single-nucleotide polymorphism.

The most significant SNP is in boldface.

Table S3. Proteins with expression levels in plasma associated with rs1166980.

**Protein**	**Beta**	**SE**	***p*-value**	**Function(s)**	**Previous association with asthma-related traits or allergic diseases** [a]
OLFML3	-0.135	0.033	4.68×10^{-5}	Protein-protein interactions, cell adhesion, intercellular interactions, early development	Yes
PCSK3	0.123	0.033	2.04×10^{-4}	Protein processing, tumor progression, virus infection	Yes
CCDC80	-0.115	0.033	4.90×10^{-4}	Cell adhesion, matrix assembly	Yes
PI3	-0.113	0.033	6.76×10^{-4}	Innate immune system	Yes
ZNF180	-0.108	0.033	1.10×10^{-3}	Transcriptional regulation; viral regulation	Yes
PPP2R1A	0.103	0.033	1.86×10^{-3}	Negative control of cell growth and division	Yes
ROR1	-0.101	0.033	2.34×10^{-3}	Development of central nervous system	NA
VIT	-0.100	0.033	2.45×10^{-3}	Cell adhesion and migration	Yes
CNTFR	-0.100	0.033	2.51×10^{-3}	Neuronal cell survival	Yes
EPHB2	-0.097	0.033	3.31×10^{-3}	Cell division and differentiation	Yes
APOE	0.161	0.055	3.39×10^{-3}	Lipoprotein metabolism	Yes
GALNT16	-0.095	0.033	3.98×10^{-3}	Metabolism of proteins	Yes
COLEC11	-0.094	0.033	4.37×10^{-3}	Innate immune system	NA
TNFRSF21	-0.091	0.033	5.75×10^{-3}	T helper cell activation, inflammation, immune regulation	NA
CLMP	-0.091	0.033	6.17×10^{-3}	Cell-cell adhesion (endothelial, epithelial), adipocyte maturation	Yes
SNRPF	0.152	0.055	6.17×10^{-3}	mRNA splicing	Yes
PMEPA1	0.090	0.033	6.46×10^{-3}	Negative regulator of TGF-β activity	Yes

[a] Proteins without available evidence of direct or indirect implication in asthma-related traits or allergic diseases are denoted by NA.

NA: not available; SE; standard error; TGF-β: transforming growth factor β.

Information provided by PhenoScanner v2.

Table S3 (continuation). Proteins with expression levels in plasma associated with rs1166980.

Protein	Beta	SE	*p*-value	Function(s)	Previous association with asthma-related traits or allergic diseases [a]
TBXAS1	-0.090	0.033	6.46×10^{-3}	Drug metabolism, synthesis of cholesterol, steroids and lipids	Yes
SCGB1D2	0.090	0.033	6.61×10^{-3}	Regulation of steroid hormones	Yes
ERAP1	-0.151	0.056	6.67×10^{-3}	Blood pressure regulation	NA
TMEM132A	-0.090	0.033	6.76×10^{-3}	Brain development	Yes
UNC5A	-0.090	0.033	6.92×10^{-3}	Axon guidance	Yes
NMES1	0.089	0.033	7.08×10^{-3}	Squamous cell carcinoma	NA
APOE3	0.148	0.055	7.18×10^{-3}	Lipoprotein metabolism	Yes
RGMB	-0.089	0.033	7.41×10^{-3}	Development of nervous system	Yes
NPPB	-0.083	0.031	7.51×10^{-3}	Blood pressure regulation	Yes
RNF149	-0.088	0.033	7.94×10^{-3}	Ligase and ubiquitin protein transferase	Yes
APOE4	0.146	0.055	8.12×10^{-3}	Lipoprotein metabolism	Yes
KLK14	-0.088	0.033	8.13×10^{-3}	Blood pressure, desquamation	Yes
EPOR	0.088	0.033	8.32×10^{-3}	Erythroblast proliferation and differentiation	Yes
SLC5A8	0.087	0.033	8.32×10^{-3}	Transport of glucose, salts, vitamins	NA
HPGD	0.087	0.033	8.51×10^{-3}	Metabolism of prostaglandins, inflammation	NA
EPHA5	0.087	0.033	8.51×10^{-3}	Developmental events	NA
MAGI2	-0.087	0.033	8.91×10^{-3}	Signaling in neuronal cells	NA
MGAT2	0.086	0.033	9.33×10^{-3}	Glycosylation	NA
LAMC2	-0.086	0.033	9.55×10^{-3}	Cell membrane	Yes
LRRC15	-0.086	0.033	9.77×10^{-3}	Collagen and laminin binding	NA
TFF3	0.086	0.033	9.77×10^{-3}	Mucosa protection	NA

[a] Proteins without available evidence of direct or indirect implication in asthma-related traits or allergic diseases are denoted by NA.

NA: not available; SE; standard error; TGF-β: transforming growth factor β.

Information provided by PhenoScanner v2.

Table S4. Proteins with expression levels affected by the SNP rs72891545.

Protein	Beta	SE	*p*-value	Function(s)	Previous association with asthma-related traits or allergic diseases [a]
GFRA1	0.522	0.148	4.07 x 10^{-4}	Neuronal cell survival and differentiation	NA
RTN4R	0.479	0.148	1.17 x 10^{-3}	Axonal regeneration	Yes
GDF11	0.453	0.148	2.19 x 10^{-3}	Ligand of TGF-β	Yes
ERAP2	0.449	0.148	2.34 x 10^{-3}	Innate immune system, generation of HLA class I binding proteins	NA
FCN1	-0.450	0.148	2.34 x 10^{-3}	Innate immune system	NA
SIGLEC9	0.450	0.148	2.34 x 10^{-3}	Innate immune system	Yes
COL8A1	-0.446	0.148	2.57 x 10^{-3}	Collagen structure	Yes
APOF	-0.444	0.148	2.69 x 10^{-3}	Transport and sterification of cholesterol	Yes
TEPSIN	0.443	0.148	2.75 x 10^{-3}	Vesicular trafficking of proteins	Yes
SLC6A16	0.439	0.148	2.95 x 10^{-3}	Neurotransmitter transporter activity	NA
PDLIM4	-0.437	0.148	3.09 x 10^{-3}	Bone development	Yes
MSTN	0.431	0.148	3.55 x 10^{-3}	Ligand of TGF-β	NA
PLG	0.422	0.148	4.37 x 10^{-3}	Degradation of plasma proteins	Yes
BARD1	-0.417	0.148	4.79 x 10^{-3}	Control of cell cycle	NA
GHR	0.404	0.148	6.31 x 10^{-3}	Growth	Yes
PLK1	0.400	0.148	6.92 x 10^{-3}	Transferase activity	NA
SNAP29	-0.395	0.148	7.59 x 10^{-3}	Membrane trafficking	Yes
LEP	0.389	0.148	8.51 x 10^{-3}	Energy homeostasis	NA
LEP	0.389	0.148	8.51 x 10^{-3}	Energy homeostasis	NA

[a] Proteins without available evidence of direct or indirect implication in asthma-related traits or allergic diseases are denoted by NA.

HLA: human leukocyte antigen; NA: not available; SE: standard error; TGF-β: transforming growth factor β.

Information provided by PhenoScanner v2.

Table S5. Genes associated with ICS response by genome-wide association studies published to date.

Genes associated	Population	Sample size	Age group	Definition of ICS response	Reference
UMAD1-GLCCI1	European	118	Children	% ΔFEV_1	1
PDE10A-T, HRH4-ZNF521	European	418	Children + adults	% ΔFEV_1	2
ALLC	Asian	189	Adults	% ΔFEV_1	3
ZNF432-ZNF841	European	581	Children	BDR	4
FBXL7	European	124	Children	Asthma symptoms	5
CMTR1,MAGI2,TRIM24, SHB-ALDH1B1, L3MBTL4-ARHGAP28, ELMO2-ZNF334	European	369	Children + adults	Asthma exacerbations	6
MMS22L-FBXL4, NAV2-HTATIP2	European	120	Adults	% ΔFEV_1	7
NA	European	110	Children	% ΔFEV_1, AHR	8
NA	Multiple (European, admixed, Asian)	2,672	Adults	% ΔFEV_1	9
EDDM3B	Admixed	244	Children + adults	ACT	10

ACT: asthma control test; AHR: airway hyperresponsiveness; BDR: bronchodilator response; ICS: inhaled corticosteroids; ΔFEV_1: change in forced expiratory volume in one second.

Citations:

1. Tantisira KG, et al. Genome-wide association between *GLCCI1* and response to glucocorticoid therapy in asthma. N Engl J Med 2011; 365:1173-1183.

2. Tantisira KG, et al. Genome-wide association identifies the *T* gene as a novel asthma pharmacogenetic locus. Am J Respir Crit Care Med 2012; 185:1286-1291.

3. Park TJ, et al. Genome-wide association study identifies *ALLC* polymorphisms correlated with FEV_1 change by corticosteroid. Clin Chim Acta 2014; 436:20-26.

4. Wu AC, et al. Inhaled corticosteroid treatment modulates *ZNF432* gene variant's effect on bronchodilator response in asthmatics. J Allergy Clin Immunol 2014; 133:723-8 e3.

5. Park HW, et al. Genetic predictors associated with improvement of asthma symptoms in response to inhaled corticosteroids. J Allergy Clin Immunol 2014; 133:664-9 e5.

6. Dahlin A, et al. *CMTR1* is associated with increased asthma exacerbations in patients taking inhaled corticosteroids. Immun Inflamm Dis 2015; 3:350-359.

7. Wang Y, et al. Pharmacodynamic genome-wide association study identifies new responsive loci for glucocorticoid intervention in asthma. Pharmacogenomics J 2015; 15:422-429.

8. Leusink M, et al. Genetic variation in uncontrolled childhood asthma despite ICS treatment. Pharmacogenomics J 2016; 16:1158-1163

9. Mosteller M, et al. No evidence of large genetic effects on steroid response in asthma patients. J Allergy Clin Immunol 2017; 139:797-803 e7.

10. Levin AM, et al. Integrative approach identifies corticosteroid response variants in diverse populations with asthma. J Allergy Clin Immunol 2019; 143:1791-1802.

Table S6. Results of SNP-level replication of previous associations of ICS response in the results of the GWAS of the change in FEV_1 after ICS treatment performed in the SLOVENIA study.

					**Published GWAS of ICS response**				**GWAS of the change in FEV_1 after ICS use (n = 166)**	
**Nearest gene(s)**	**SNP**	**Chr.[a]**	**Position[b]**	**A1/A2**	**Definition of ICS response**	**OR (95% CI)[c]**	***p*-value**	**Citation**	**OR (95% CI)[c]**	***p*-value**
ALLC	rs17445240	2	3703041	G/A	% ΔFEV_1	1.43 (1.25-1.65)	5.01×10^{-7}	1	1.23 (0.62-2.43)	0.555
	rs13418767	2	3704830	T/G	% ΔFEV_1	1.40 (1.22-1.62)	2.77×10^{-6}		1.11 (0.57-2.16)	0.753
	rs6754459	2	3707423	T/C	% ΔFEV_1	1.43 (1.24-1.65)	5.73×10^{-7}		1.32 (0.81-2.16)	0.268
	rs17017879	2	3713658	C/G	% ΔFEV_1	1.40 (1.22-1.61)	2.49×10^{-6}		1.12 (0.53-2.36)	0.766
	rs7558370	2	3714261	C/A	% ΔFEV_1	1.39 (1.21-1.60)	3.73×10^{-6}		1.16 (0.60-2.23)	0.660
	rs11123610	2	3723026	A/G	% ΔFEV_1	0.69 (0.60-0.80)	3.57×10^{-7}		0.78 (0.49-1.24)	0.287
FBXL7	rs10044254	5	15783596	G/A	Asthma symptoms	3.29 (1.94-5.58)	1.02×10^{-5}	2	0.84 (0.50-1.42)	0.511
CMTR1	rs2395672	6	37428577	G/A	Asthma exacerbations	1.08 (1.04-1.12)	1.86×10^{-5}	3	1.78 (1.03-3.05)	**0.037**
MMS22L-FBXL4	rs6924808	6	98358575	A/G	% ΔFEV_1	NA	5.31×10^{-7}	4	0.95 (0.61-1.50)	0.841
PDE10A-T	rs6456042	6	166534742	C/A	% ΔFEV_1	NA	6.67×10^{-6}	5	0.78 (0.49-1.23)	0.282
	rs3127412	6	166535561	T/C	% ΔFEV_1	NA	9.68×10^{-6}		0.78 (0.49-1.23)	0.282
	rs1134481	6	166571164	G/T	% ΔFEV_1	NA	NA		0.87 (0.56-1.35)	0.524
	rs2305089	6	166579270	T/C	% ΔFEV_1	NA	NA		0.86 (0.54-1.36)	0.520
	rs3099266	6	166581147	C/T	% ΔFEV_1	NA	NA		0.94 (0.60-1.48)	0.801

[a]Chromosome; [b]Positions based on GRCh37/hg19 build; [c]Odds ratio for the effect alleles.

A1: Effect allele; A2: Non-effect allele; ACT: asthma control test; BDR: bronchodilator response; CI: Confidence Interval; GWAS: genome-wide association study; ICS: inhaled corticosteroids; NA: not available; SNP: single-nucleotide polymorphism; ΔFEV_1: change in forced expiratory volume in one second. SNPs with evidence of replication in European populations are in boldface.

Citations:

1. Park TJ, *et al.* Genome-wide association study identifies *ALLC* polymorphisms correlated with FEV_1 change by corticosteroid. Clin Chim Acta 2014; 436:20-26.

2. Park HW, *et al.* Genetic predictors associated with improvement of asthma symptoms in response to inhaled corticosteroids. J Allergy Clin Immunol 2014; 133:664-9 e5.

3. Dahlin A, *et al. CMTR1* is associated with increased asthma exacerbations in patients taking inhaled corticosteroids. Immun Inflamm Dis 2015; 3:350-359.

4. Wang Y, *et al.* Pharmacodynamic genome-wide association study identifies new responsive loci for glucocorticoid intervention in asthma. Pharmacogenomics J 2015; 15:422-429.

5. Tantisira KG, *et al.* Genome-wide association identifies the *T* gene as a novel asthma pharmacogenetic locus. Am J Respir Crit Care Med 2012; 185:1286-1291.

Table S6 (continuation). Results of SNP-level replication of previous associations of ICS response in the results of the GWAS of the change in FEV_1 after ICS treatment performed in the SLOVENIA study.

					**Published GWAS of ICS response**				**GWAS of the change in FEV_1 after ICS use (n = 166)**	
**Nearest gene(s)**	**SNP**	**Chr.** [a]	**Position** [b]	**E/NE**	**Definition of ICS response**	**OR (95% CI)** [c]	***p* – value**	**Citation**	**OR (95% CI)** [c]	***p* – value**
UMAD1-GLCCI1	rs37972	7	8007509	C/T	% ΔFEV_1	NA	0.010	6	0.97 (0.63 – 1.48)	0.876
MAGI2	rs2691529	7	77803275	T/C	Asthma exacerbations	0.97 (0.94 – 1.00)	0.051	3	1.01 (0.57 – 1.81)	0.966
TRIM24	rs6467778	7	138178222	G/A	Asthma exacerbations	1.01 (1.00 – 1.03)	0.021	3	0.67 (0.39 – 1.16)	0.151
SHB-ALDH1B1	rs4271056	9	38232043	C/T	Asthma exacerbations	0.96 (0.93 – 0.99)	6.71×10^{-3}	3	1.16 (0.66 – 2.03)	0.615
NAV2-HTATIP2	rs1353649	11	20253599	G/A	% ΔFEV_1	NA	3.92×10^{-9}	4	0.83 (0.51 – 1.35)	0.444
EDDM3B	rs3827907	14	21238798	C/T	ACT	NA	7.79×10^{-8}	7	0.52 (0.32 – 0.84)	$\mathbf{7.40 \times 10^{-3}}$
L3MBTL4-ARHGAP28	rs9303988	18	6667583	C/T	Asthma exacerbations	1.03 (1.00 – 1.05)	0.012	3	1.30 (0.81 – 2.11)	0.279
HRH4-ZNF521	rs9955411	18	22074720	T/A	% ΔFEV_1	NA	1.28×10^{-4}	5	0.94 (0.56 – 1.58)	0.814
ZNF432-ZNF841	rs3752120	19	52552021	T/C	BDR	1.03 (1.02 – 1.05)	4.58×10^{-6}		0.97 (0.55 – 1.71)	0.917
	rs3450	19	52552999	C/T	BDR	1.03 (1.02 – 1.04)	1.93×10^{-6}	8	0.89 (0.52 – 1.54)	0.681
	rs12460587	19	52586919	G/T	BDR	1.04 (1.02 – 1.05)	5.69×10^{-7}		0.98 (0.56 – 1.73)	0.948
ELMO2-ZNF334	rs279728	20	45080421	T/C	Asthma exacerbations	1.02 (1.01 – 1.03)	6.45×10^{-3}	3	2.59 (0.96 – 6.99)	0.061

[a] Chromosome; [b] Positions based on GRCh37/hg19 build; [c] Odds ratio for the effect alleles.

ACT: asthma control test; BDR: bronchodilator response; CI: Confidence Interval; E: Effect allele; GWAS: genome-wide association study; ICS: inhaled corticosteroids; NA: not available; NE: Non-effect allele; SNP: single-nucleotide polymorphism; ΔFEV_1: change in forced expiratory volume in one second.

Citations:

6. Tantisira KG, *et al.* Genome-wide association between *GLCCI1* and response to glucocorticoid therapy in asthma. N Engl J Med 2011; 365:1173-1183.

7. Levin AM, *et al.* Integrative approach identifies corticosteroid response variant in diverse populations with asthma. J Allergy Clin Immunol 2019;143:1791-1802.

8. Wu AC, *et al*. Inhaled corticosteroid treatment modulates *ZNF432* gene variant's effect on bronchodilator response in asthmatics. J Allergy Clin Immunol 2014; 133:723-8 e3.

Significant *p*-values are in boldface.

Table S7. Genomic-region replication of previous associations of ICS response. Evidence of association with the ΔFEV_1 after ICS treatment in asthmatic patients.

Gene	# SNPs tested	# Independent signals	Bonferroni *p*-value threshold	Significant SNPs after Bonferroni-like correction	SNP min *p*-value	A1/A2	OR (95% CI)[a]	*p*-value
ALLC	970	112	4.48×10^{-4}	NA	rs11538545	C/G	1.20 (0.71-2.05)	0.496
FBXL7	1251	69	7.19×10^{-4}	NA	rs1019810	G/A	0.46 (0.28-0.77)	2.88×10^{-3}
CMTR1	588	31	1.61×10^{-3}	NA	rs56242039	C/G	0.30 (0.14-0.67)	3.40×10^{-3}
MMS22L-FBXL4	4218	100	5.01×10^{-4}	NA	rs1206129	A/G	0.50 (0.31-0.80)	3.77×10^{-3}
PDE10A-T	3872	138	3.63×10^{-4}	rs9365939	rs9365939	G/A	0.41 (0.26-0.65)	$\mathbf{1.92 \times 10^{-4}}$
				rs2118353	rs2118353	T/C	0.41 (0.26-0.65)	$\mathbf{1.92 \times 10^{-4}}$
UMAD1-GLCCI1	2508	196	2.55×10^{-4}	NA	rs200043140	A/G	0.23 (0.10-0.52)	3.98×10^{-4}
MAGI2	5798	415	1.21×10^{-4}	NA	rs17417090	C/T	2.51 (1.44-4.37)	1.20×10^{-3}
TRIM24	796	29	1.74×10^{-3}	NA	rs17837468	G/A	0.40 (0.19-0.86)	0.018
SHB-ALDH1B1	2009	18	2.75×10^{-3}	NA	rs3808862	A/G	1.99 (1.25-3.15)	3.42×10^{-3}
				NA	rs7047650	C/G	1.99 (1.25-3.15)	3.42×10^{-3}
				NA	rs4878179	T/C	1.99 (1.25-3.15)	3.42×10^{-3}
				NA	rs10814650	G/A	1.99 (1.25-3.15)	3.42×10^{-3}
				NA	rs10814651	T/C	1.99 (1.25-3.15)	3.42×10^{-3}

[a]Odds ratio for the effect alleles. A1: Effect allele; A2: Non-effect allele; CI: Confidence Interval; ICS: inhaled corticosteroids; NA: not available; SNP: single-nucleotide polymorphism; ΔFEV_1: change in forced expiratory volume in one second.

Significant *p*-values after multiple comparison adjustment are in boldface.

Table S7 (continuation). Genomic-region replication of previous associations of ICS response. Evidence of association with the ΔFEV_1 after ICS treatment in asthmatic patients.

Gene	# SNPs tested	# Independent signals	Bonferroni *p*-value threshold	Significant SNPs after Bonferroni-like correction	SNP min *p*-value	A1/A2	OR (95% CI)[a]	*p*-value
NAV2-HTATIP2	2694	114	4.36 x 10^{-4}	NA	rs73429709	C/T	2.55 (1.42-4.57)	1.63 x 10^{-3}
EDDM3B	912	84	5.97 x 10^{-4}	NA	rs57736800	G/A	0.49 (0.30-0.80)	4.17 x 10^{-3}
				NA	rs61552195	C/T	0.49 (0.30-0.80)	4.17 x 10^{-3}
L3MBTL4-ARHGAP28	3434	135	3.70 x 10^{-4}	NA	rs8083583	T/C	0.45 (0.27-0.78)	4.11 x 10^{-3}
HRH4-ZNF521	2983	147	3.39 x 10^{-4}	NA	rs11877115	C/T	2.34 (1.24-4.42)	8.43 x 10^{-3}
ZNF432- ZNF841	875	57	8.76 x 10^{-4}	NA	rs111463681	T/C	3.33 (1.36-8.13)	8.42 x 10^{-3}
ELMO2-ZNF334	709	83	6.02 x 10^{-4}	NA	rs76086573	C/A	0.12 (0.03-0.58)	8.03 x 10^{-3}

[a]Odds ratio for the effect alleles. A1: Effect allele; A2: Non-effect allele; CI: Confidence Interval; ICS: inhaled corticosteroids; NA: not available; SNP: single-nucleotide polymorphism; ΔFEV_1: change in forced expiratory volume in one second.

Significant *p*-values after multiple comparison adjustment are in boldface.

Journal of Personalized Medicine

Supplementary figure legends

Figure S1. Quantile-quantile plot of association results of ICS response measured as the binary outcome related to the change in FEV_1 after ICS treatment. The logarithmic transformation of the observed and expected association results ($-\log_{10}$ *p*-value) is represented on the *y*-axis and *x*-axis, respectively ($\lambda_{GC} = 1.00$).

Figure S2. Regional plot of association results with asthma exacerbations despite ICS use in European children and young adults. The *y*-axis represents the logarithmic transformation of the association results ($-\log_{10}$ *p*-value) by chromosome position (*x*-axis) for each SNP as a dot. The most significant variant after Bonferroni-like correction is represented by a diamond (rs72891545). The remaining SNPs are color-coded based on pairwise linkage disequilibrium (r^2 values) with that SNP for European populations from 1KGP (GRCh37/hg19 build).

Figure S1

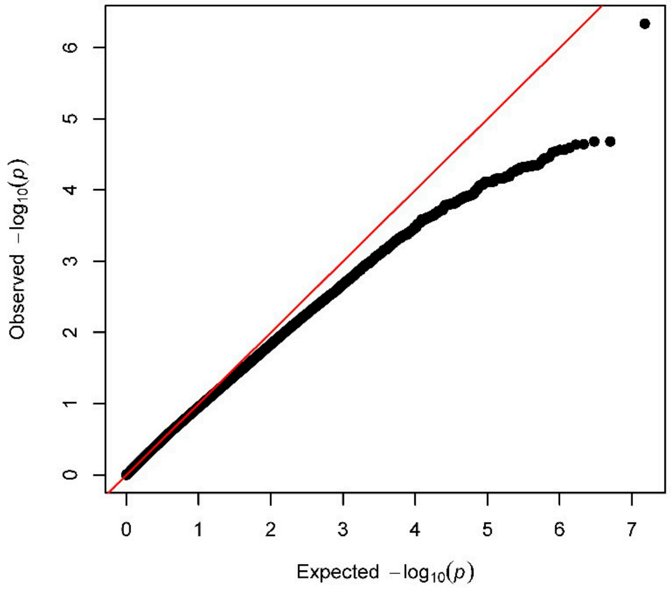

Figure S2

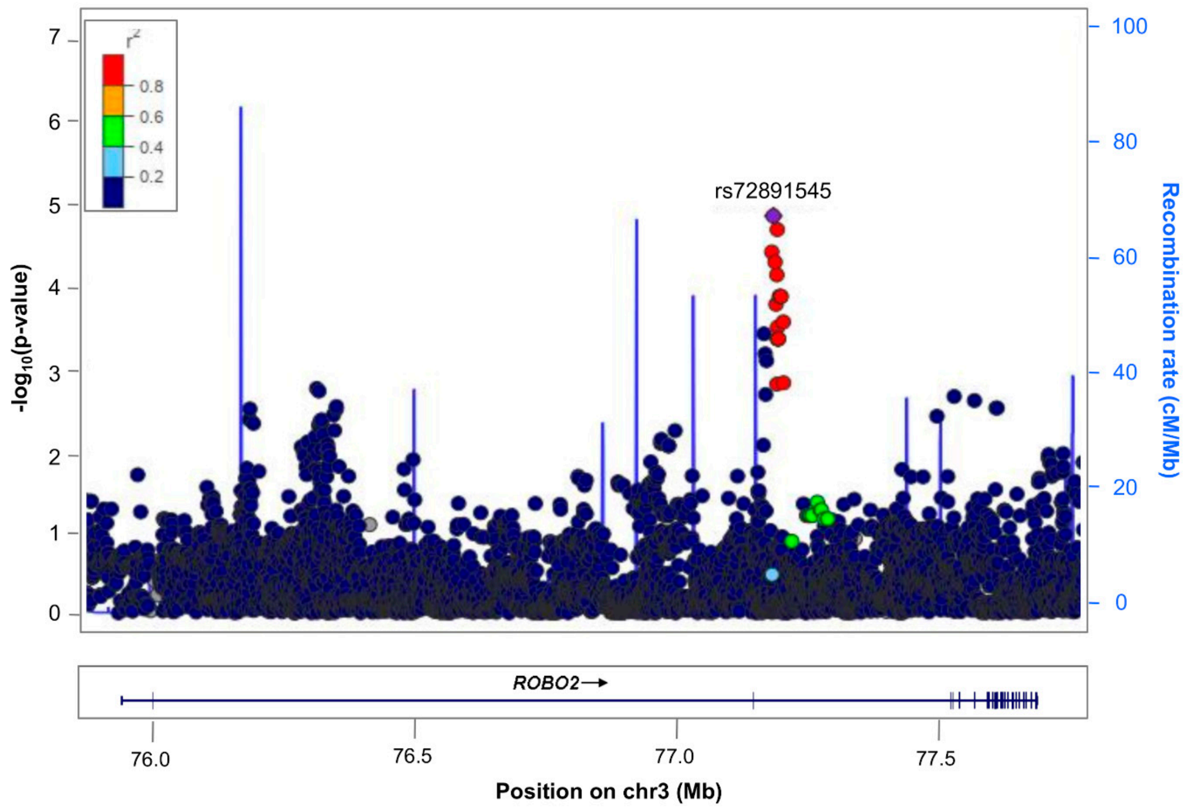
rs72891545
r²
0.8
0.6
0.4
0.2
-log10(p-value)
Recombination rate (cM/Mb)
ROBO2→
76.0
76.5
77.0
77.5
Position on chr3 (Mb)
